# Identification of broadly-conserved parasitic nematode proteins that activate immunity

**DOI:** 10.3389/fpara.2023.1223942

**Published:** 2023-08-08

**Authors:** Bruce A. Rosa, Dante S. Zarlenga, Valsin M. Fournet, Ethiopia Beshah, Dolores E. Hill, Alexander Zarlenga, Angela Yee, Xiaowu Liang, Adam D. Shandling, Amit Oberai, Joseph F. Urban, Makedonka Mitreva

**Affiliations:** ^1^ Division of Infectious Diseases, Department of Medicine, Washington University School of Medicine, St. Louis, MO, United States; ^2^ U.S. Department of Agriculture, Northeast Area, Agricultural Research Service, Beltsville Agricultural Research Center, Animal Parasite Diseases Laboratory and Beltsville Human Nutrition Research Center, Diet Genomics and Immunology Laboratory, Beltsville, MD, United States; ^3^ Antigen Discovery Inc. (ADI) ImmPORT Therapeutics Inc., Irvine, CA, United States; ^4^ Department of Genetics, Washington University School of Medicine in St. Louis, St. Louis, MO, United States; ^5^ McDonnell Genome Institute, Washington University in St. Louis, St. Louis, MO, United States

**Keywords:** nematode, *Ascaris suum*, *Trichuris suis*, roundworm, whipworm, protein array, immunity

## Abstract

**Introduction:**

Soil transmitted nematodes are impediments to human health and agricultural production. Poor anthelmintic efficiencies, the emergence of resistant strains, and the persistence of infective stages highlight the need for more effective control strategies. Parasitic nematodes elicit a Th2-type immune response that most often is not protective. Vaccination has thus far been unsuccessful due to unrealized antigenic characters and unknown mechanisms that nematodes use to circumvent host immunity.

**Methods:**

Here, we used a genomics/proteomics approach (including immunoblot experiments from pigs infected with *T. suis*) to prioritize putative immunogenic excretory/secretory (E/S) proteins conserved across and specific to several gastrointestinal (GI) parasitic nematode species. A cocktail of five recombinant proteins optimized for conserved GI nematode targets was used immunize pigs and test for active antibody responses in both the serum and intestinal ileal fluid of immunized pigs. An antibody-protein array of putative immunogenic proteins was developed from a combined bioinformatic, experimental, and literature-based prioritization of homologous parasite proteins.

**Results:**

Screening the array with sera and ileal fluid samples from immunized pigs suggested cross-reactivity among homologous proteins and a general activation of immunity. PCA clustering showed that the overall immune responses were altered by immunization, but no substantial changes were observed following direct worm challenge with either *Ascaris suum* or *Trichuris suis*.

**Discussion:**

Proteins that activated immunity are potential antigens for immunization and the multi-omics phylum-spanning prioritization database that was created is a valuable resource for identifying target proteins in a wide array of different parasitic nematodes. This research strongly supports future studies using a computational, comparative genomics/proteomics approach to produce an effective parasite vaccine.

## 1 Introduction

Parasites belonging to the phylum Nematoda cause numerous diseases and economic loss in humans, animals, and plants. These place major burdens on human health and agricultural production resulting from morbidity, mortality, the cost of treatment, and implementation of control programs. Soil transmitted nematodes i.e., *Ascaris lumbricoides*, *Trichuris trichiura* and the hookworms Necator americanus and Ancylostoma duodenale, infect 1.5 billion people worldwide, accounting for 1.9 million Disability Adjusted Life Years (DALYs), and causing anemia, malnutrition, educational loss, and cognitive deficits (WHO, 2022). Current methods used to control or reduce the impact of nematode infections rely heavily on anthelmintics including plant nematicides. Although short-comings of chemical-based methods are well recognized, the general approach has provided enormous benefits to human health and agricultural production; consequently, the use of anthelmintics is likely to remain a major factor in integrated methods of parasite control. However, deficiencies of current anthelmintics and control measures include: 1) the increasing and widespread occurrence of nematode strains with selection for anthelmintic resistance (Fissiha and Kinde, 2021); 2) repeated occupational exposure and environmental impact presented by anthelmintics and their residues (Beynon, 2012; Skuce et al., 2022); 3) the relatively poor efficacy of available anthelmintics against some nematode pathogens (Moser et al., 2017); 4) the unlikely eradication of these pathogens given their diverse genetic make-up, 5) inadequate investment in public health for populations most at risk, and 6) the omnipresence of some species in wildlife and, in many cases, multi-host life-cycles that act as reservoirs for deleterious parasite genotypes.

There is a clear need to identify better anthelmintics that address the shortcomings of those currently available given the potential benefits. However, the problem of acquired resistance to anthelmintics by nematodes has surfaced in nearly all drugs currently available and is likely to persist given the high levels of genetic diversity among this group of pathogens, the often-inappropriate dosing, and the suboptimal mode of application to many infected hosts. The integration of complementary control programs along with anthelmintic treatment can provide additive or synergistic benefits while prolonging the effectiveness of each individual method. Of particular interest are technologies that exploit host-based or indirect methods of control e.g., acquired (animal) or innate (animals and plants) immunity to parasitic nematodes.

Proteins that are excreted or secreted (E/S) by the parasite can activate innate and/or acquired immunity and are also involved in the induction and/or maintenance of the disease state through immune modulating properties. Identifying and characterizing proteins released during the parasitic stages is crucial to addressing many of the unmet needs of diagnosing and treating these clinically and economically important parasites. Further, this will assist in advancing a better understanding of the biology of nematodes. Parasitic nematode infections are featured as a chronic disease without sterile immunity wherein the worms can live within the host for many years (John and Petri, 2006). It is well accepted that long periods of survival in the host result from parasite immune-modulating and immune-evasive capacities (Hewitson et al., 2009) that can also minimize severe host pathology (Behnke et al., 1992; Maizels and Yazdanbakhsh, 2003; Summers et al., 2005). One proposed mechanism of modulation is through the release of soluble mediators that degrade or interact with host immune regulatory cells and molecules (Lightowlers and Rickard, 1988). A strategy to identify immunogenic proteins from parasitic larval stages prior to the development of immune modulating adult infections is supported by several examples of protective immunity induced by attenuated live vaccines (Cornwell and Jones, 1970; Urban and Tromba, 1984; Chapman et al., 2021).

Nematodes predominantly elicit a Th2-type immune response during infection that often does not quickly render the host refractory to re-infection. This is particularly evident in *Ostertagia ostertagi* infections of cattle, where years of host re-exposure to the parasite are required before a meaningful protective response is generated. To date, vaccination against nematodes with naturally-derived antigens has been difficult to achieve given the inability to culture these organisms through the five stages of development and purify the proteins (Claerebout et al., 2003; Vercauteren et al., 2004). In the case of *Haemonchus contortus*, recombinant antigens have largely been superseded by natural antigens derived from adult worms (Adduci et al., 2022); however, ethical issues have arisen because live animals are required to obtain enough worms for antigen production which also limits global distribution of the vaccine. In contrast, few examples exist where recombinant antigens have been used successfully to vaccinate against extracellular metazoan parasites i.e., cestodes (Lightowlers, 2004; Gauci et al., 2005).

Several examples of host immune modulation by parasite E/S products have been identified including: 1) macromolecules that share interferon gamma (IFN-γ) epitopes (Grencis and Entwistle, 1997); 2) macrophage migration-inhibitory factors that affect macrophage maturation (Pennock et al., 1998); 3) molecules that bind Toll-like receptors (TLRA-4) and down-regulate Th2 responses (Helmby and Grencis, 2003); 4) reduced mucosal allergic inflammation and interference with dendritic cell function (McConchie et al., 2006) and; 5) immune suppression in swine (Souza et al., 2002) and cattle (Gomez-Munoz et al., 2004). When parasite adaptation is coupled with the large genetic variability both within and between worm populations (as well as the genetic diversity of the host) the development of long term, unilateral treatments to attenuate parasite growth, development and survival presents a challenge. Nevertheless, antigens derived from parasitic nematodes have been shown to induce protective immunity (Urban and Romanowski, 1985; Newton and Munn, 1999) but the relevant physical characteristics of those antigens have yet to be identified. A deeper understanding of the antigenic characteristics and the mechanisms utilized by nematodes to survive host immune responses would lead to innovative methods of vaccination.

The current availability of detailed “multi-omics” datasets for parasitic helminths has opened new opportunities for the prioritization and identification of vaccine targets for infection control (Vrushabh et al., 2022). However, current studies primarily rely on proteomics datasets, with fewer utilizing transcriptomic and genomic data, and no described multi-omics studies for helminth vaccine targets have been described that have successful follow-up with *in vivo* studies (Vrushabh et al., 2022). To this end, we have used a computational, comparative genomics/transcriptomics/proteomics approach to seek out putative immunogenic E/S proteins conserved among and specific to GI nematodes, since these are the most likely to be crucial for their survival in their GI niche. The approach utilizes a bioinformatic approach as well as experimental sera-based infection results to optimize and prioritize widely-conserved GI nematode targets in a comprehensive manner. Further, active antibody responses to a selected vaccination cocktail and subsequent challenge with nematode infections (*Ascaris suum* infection in pigs) were quantified using an antibody-protein array containing both the target vaccination proteins as well as other proteins expected to be immunogenic in the hosts based on an additional bioinformatic, proteomic, and literature-based prioritization. *Ascaris suum* serves as an excellent model for the human roundworm *Ascaris lumbricoides*, since *A. suum* can infect humans and the two species are so closely related that some researchers consider them to be the same species (Leles et al., 2012). Furthermore, the pig model has many advantages to serve as an animal model for human diseases, including its very high similarities to humans in anatomy and immune system functions (e g., the presence of tonsils), with the porcine immune system resembling humans for more than 80% of analyzed parameters (in contrast to only about 10% in mice) (Pabst, 2020). Because of this, and because of the bioinformatic prioritization and experimental results favoring targets that are broadly conserved across GI nematodes (including with *Trichuris suis*, very closely related to the human whipworm *Trichuris trichiura* (Cutillas et al., 2009 *)*), the results from the animal experiments presented here are expected to be applicable for future vaccination trials against parasitic helminths in humans, as well as to animals of veterinary importance.

## 2 Results / discussion

### 2.1 Overall experimental approach

**Figure 1:**
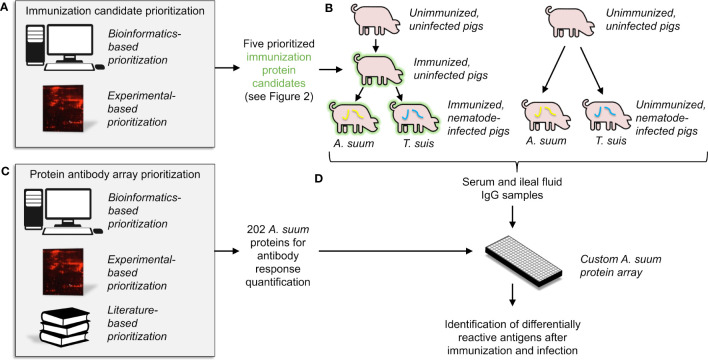
An overview of the overall experimental approach, which includes **(A)** the computational and experimental-based prioritization of immunization protein candidates, **(B)** experimental immunization of the prioritized candidates into pigs, with serum and intestinal ileal fluid samples collected, **(C)** computational and experimental-based prioritization of 202 proteins for use in the antibody-protein array, and **(D)** statistical analysis of detected antibodies based on results from the antibody-protein array.

With the goal of identifying and validating putative nematode-conserved immunogenic E/S proteins, our overall experimental approach included: (i) prioritization of five vaccination candidates for experimental validation, using both bioinformatic and experimental evidence (**Figure 1A**), (ii) immunization of pigs with the prioritized immunogenic E/S protein cocktail, followed by infection with two different nematode species (*Ascaris suum* and *Trichuris suis*) (**Figure 1B**), (iii) prioritization of 202 predicted nematode immunogenic proteins for the construction of an antibody-protein array to detect other candidate proteins reactive with sera from immunized and parasite infected animals (**Figure 1C**) and (iv) validation of antibody responses to the immunogenic E/S proteins and additional homologous proteins in samples from both the sera and intestinal ileal fluids of immunized and experimentally infected pigs (**Figure 1D**).

### 2.2 Clustering and filtering of orthologous protein families

The genomes of four representative GI nematode species of importance to animal and human health (O'connor et al., 2006) spanning the phylum Nematoda (clades I to V (Blaxter et al., 1998)) were used to prioritize vaccine targets for testing in experimental models: *Trichuris suis* (Jex et al., 2014) (clade I) and *A. suum* (Jex et al., 2011) (clade III) (nematode parasites represented in swine and related species in humans), *H. contortus* (Laing et al., 2013) (clade V) (a nematode parasite represented in numerous ruminant hosts) and *Heligmosomoides polygyrus bakeri* (International Helminth Genomes Consortium, 2019) (clade V) (a nematode parasite represented in experimental mouse models, facilitating future experimentation using mouse infections rather than pigs). In addition to these four GI nematode species, three other non-GI nematode species (*Caenorhabditis elegans* (Harris et al., 2013)*, Dictyocaulus viviparus* (McNulty et al., 2016), *and Onchocerca ochengi* (Howe et al., 2017)) were included to distinguish GI-nematode specific proteins. Genomes for relevant host species were obtained from ENSEMBL and GenBank (Harris et al., 2013; Sayers et al., 2023) and included *Homo sapiens* for medical importance, *Canis familiaris* for veterinary importance, and *Bos taurus, Canis familiaris, Mus musculus, Ovis aries*, and *Sus scrofa* because they are host species for the parasitic helminths used (host specificity indicated in **Supplementary Table S1**). Two outgroups (*Drosophila melanogaster* and *Saccharomyces cerevisiae*, retrieved from ENSEMBL (Cunningham et al., 2022)) were included in order to distinguish universal eukaryote-conserved proteins from proteins conserved just among hosts and parasitic worms. The analysis resulted in the identification of 25,234 unique Orthologous Protein Families (OPFs) using OrthoMCL (Li et al., 2003; Fischer et al., 2011). Genome versions, protein counts, and sources are provided in **Supplementary Table S1**. Nematode proteins across all species were categorized as putatively secreted if they contained no transmembrane domains (as predicted by Phobius (Kall et al., 2004)), and contained either a signal peptide for secretion (defined by Phobius) or a non-classical secretion peptide (defined by SecretomeP 1.0 (Bendtsen et al., 2004)), the same approach used for the International Helminth Genomes Consortium study (International Helminth Genomes Consortium, 2019).

### 2.3 Computational immunogenic OPF prioritization

**Figure 2:**
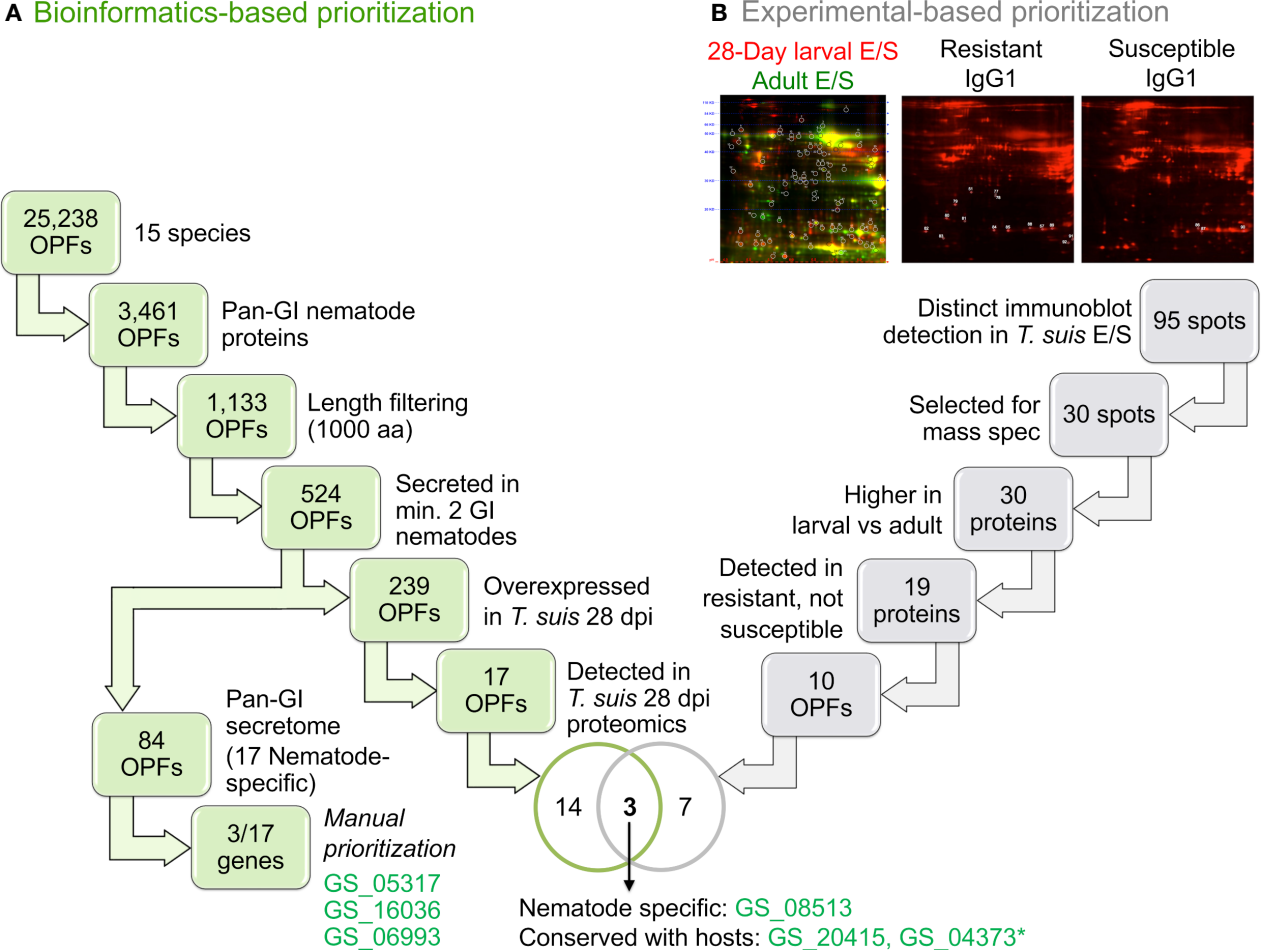
An overview of the orthologous protein family (OPF) prioritization process to identify proteins for immunization. Workflows are shown for **(A)** The bioinformatics-based prioritization approach and **(B)** the experimental-based prioritization approach. *GS_04373 was not able to be successfully cloned for the downstream experimentation.

OPFs were prioritized based on a number of criteria (**Figure 2**; more detailed summary provided in **Supplementary Figure S1**). Overall, 39.4% of GI nematode proteins were predicted to be secreted. This is comparable to (but slightly higher than) the 32% and 32.7% previously reported for *C. elegans* (Suh and Hutter, 2012) and *N. americanus* (Tang et al., 2014), respectively. This higher value may be due to better coverage resulting from the use of multiple prediction algorithms, and/or due to an expansion in the secretome of specific species (e.g., 46.7% of *H. polygyrus bakeri* genes are predicted to be secreted).

Of the 25,234 total OPFs, 14,360 contained at least one nematode protein, 3,461 of which contained at least one ortholog in each of the four GI nematodes (“conserved GI nematode OPFs”). Of those, 1,133 contained GI nematode proteins with an average gene length of less than 1000 bp to simplify downstream cloning, expression, and testing (long proteins are generally more difficult to express as soluble proteins in bacterial hosts). Of the 1,133 conserved-GI nematode OPFs filtered for length, 84 contained only GI nematode proteins which were predicted to be secreted (i.e., the “conserved-GI nematode secretome”; **Figure 2A**). These OPFs were further reduced to 17 which were nematode-specific (and an additional 67 which had orthologs in at least one host and/or out-group species). Seven of the 18 A*. suum* proteins belonging to these OPFs were overexpressed in the *A. suum* head relative to the other tissues (enrichment *P* = 6x10^-5^, binomial distribution test; **Table 1**, **Supplementary Table S2**), which is consistent with the previous identification of many nematode-specific functions being highly expressed in this tissue (Rosa et al., 2014). It should be noted that the existence of host orthologs does not necessarily equate to a poor vaccine target since indels and variations in sequence can alter antigenicity, but the proteins among the selected 17 OPFs represented the highest-priority targets.

**Table 1:** Annotations and *A. suum* transcriptomic/proteomic results for the 17 nematode-specific computationally prioritized OPFs.

OPF ID	*A. suum* gene member(s)	Putative Function (KEGG, IPR)	*A. suum* proteomics detection (Rosa et al., 2015)	*A. suum* tissue overexpression (Rosa et al., 2014)
4GI_OPF_8504	GS_16036	NADH dehydrogenase	–	Head
4GI_OPF_10967	GS_06993	Globin/Oxygen transport	Intestinal Tissue	Head
4GI_OPF_7517	GS_05317	–	–	Head
4GI_OPF_7637	GS_12274	–	Intestinal Tissue	Head
4GI_OPF_8573	GS_10125	Sialin (Sialic acid transport)	–	Head, Uterus
4GI_OPF_8551	GS_08909	–	–	Head, Ovary
4GI_OPF_8799	GS_01990	–	–	Head
4GI_OPF_7623	GS_20993	Pyruvate dehydrogenase subunit	–	Ovary
4GI_OPF_7011	GS_05184, GS_07274	–	–	Ovary
4GI_OPF_8725	GS_06822	FMRFamide-related peptide (neuropeptide)	–	Uterus
4GI_OPF_8772	GS_10140	Globin/Oxygen transport	–	Uterus
4GI_OPF_8801	GS_05200	–	–	Uterus
4GI_OPF_11020	GS_02552	IQ motif, EF-hand binding	–	–
4GI_OPF_8557	GS_15338	ALX homeobox protein 1	–	–
4GI_OPF_7658	GS_11879	basic region leucine zipper transcription factor	–	–
4GI_OPF_8600	GS_13493	–	–	–
4GI_OPF_7679	GS_05535	–	–	–

### 2.4 Experimental validation of three bioinformatics-prioritized targets

Of the 17 prioritized vaccine candidate OPFs (**Table 1**), three were selected for experimental vaccinations against two of the four GI nematode species (*A. suum* and *T. suis*), as well as *Trichinella spiralis* which was tested to demonstrate broad vaccination potential against parasitic nematode species not included in the prioritization criteria. Final OPF selection was based on both functional annotations (Interpro (Jones et al., 2014) and KEGG (Kanehisa et al., 2012)), as well as differential expression levels in both *T. suis* and *A. suum* (Rosa et al., 2014). In addition, proteomics data from a study of the *A. suum* intestine (Rosa et al., 2015) was used to determine whether OPFs were detected in specific compartments of the nematode intestine. Three OPFs were prioritized (described below), and their immunogenic potential was evaluated using ELISA testing for antibody production against the *A. suum* protein orthologs (GS_06993, GS_05317 and GS_16036) in pigs infected with *A. suum*, *T. suis* or *T. spiralis* (see methods). PCR primers used for cloning these three proteins are provided in **Supplementary Table S3**.

**Figure 3:**
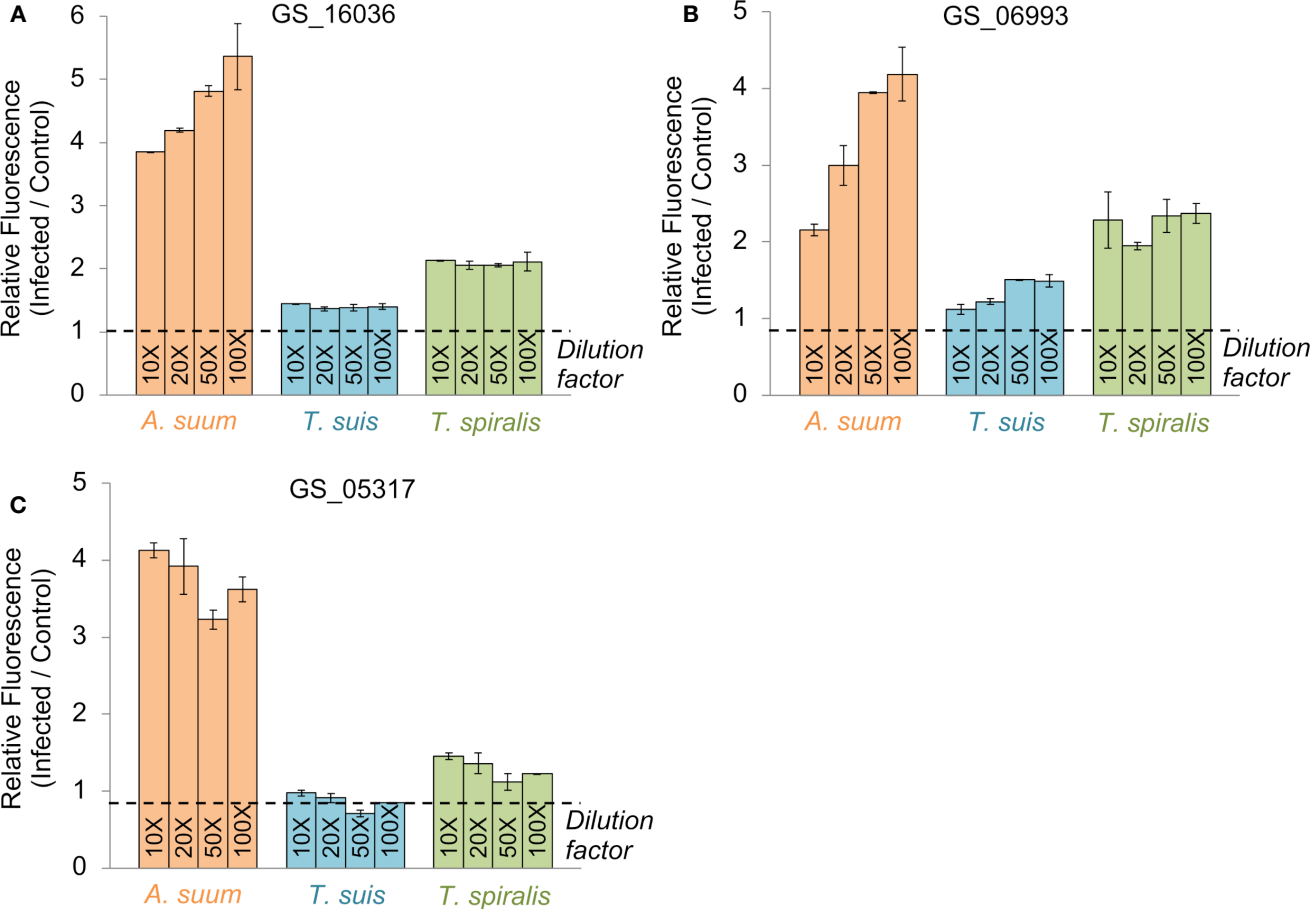
ELISA testing results for each of the three vaccine candidate proteins prioritized by the bioinformatics approach **(A)** GS_16036, **(B)** GS_06993, **(C)** GS_05317. Results are shown for four dilutions of antigen exposed to swine infection sera from pigs infected with *A. suum* (a pool of sera from three pigs given a primary and secondary infection with *A. suum*), *T. spiralis* (a pool of sera from three pigs at 60 days after a primary infection with *T. spiralis*) or *T. suis* (a pool of sera from three pigs that had cleared an adult *T. suis* worm infection at 53 days after inoculation with infective eggs). All values were calculated relative to an uninfected control sample. Error bars represent the standard deviation of two technical replicates.

The first prioritized vaccine candidate was GS_16036, belonging to 4GI_OPF_8504, and annotated as a NADH dehydrogenase (ubiquinone) 1 alpha subcomplex 7 (K03951). This was an attractive vaccination target due to its critical function in energy metabolism and its well-conserved nature among helminths (as demonstrated by its use as a marker gene for Cestode evolution (Gasser et al., 1999)). This OPF was also conserved across all three nematode outgroups (*O. ochengi*, *C. elegans*, and *D. viviparus*) and overexpressed in the *A. suum* head relative to the other tissues (Rosa et al., 2014). The ELISA experimental results indicated antibody reactivity for GS_16036 in *A. suum* as well as the phylogenetically distinct species *T. spiralis* (which was not used in the prioritization process), compared to control samples (**Figure 3A**), with a weaker response observed for *T. suis*.

The second prioritized vaccine candidate was GS_06993, belonging to 4GI_OPF_10967, and annotated as a Globin-like protein (IPR009050) involved in heme binding (GO:0020037) and oxygen transport (GO:0015671). Both these functions are attractive targets for nematode control by reducing oxygen availability during parasitism. A critical role in parasitism is further supported because the OPF is conserved among the two parasitic nematode outgroups (*O. ochengi* and *D. viviparus*) but not *C. elegans*. The GS_06993 gene is overexpressed in the *A. suum* head (Rosa et al., 2014), but its protein product was also detected in the *A. suum* intestinal lumen (Rosa et al., 2015), suggesting it may originate in the head and then pass to the intestinal tract. Cross-reactivity to GS_06993 was observed in the serum of *A. suum* and *T. spiralis-*infected pigs, with a weak signal from *T. suis*-infected pigs. (**Figure 3B**).

Finally, the third prioritized vaccine candidate was GS_05317, belonging to 4GI_OPF_7517. Like the other two prioritized candidates, this gene was overexpressed in *A. suum* adult worms, but the *T. suis* ortholog (D918_01659) was also overexpressed in the larval stages, making it a particularly interesting target for these two parasite species. More than three-fold increase in antibody binding was observed in ELISA for *A. suum*, while *T. spiralis* exhibited weak cross-reactivity as well. These data support the concept of a conserved nematode vaccine target despite a lack of reactivity with sera from *T. suis* infected pigs (**Figure 3C**).

**Figure 4:**
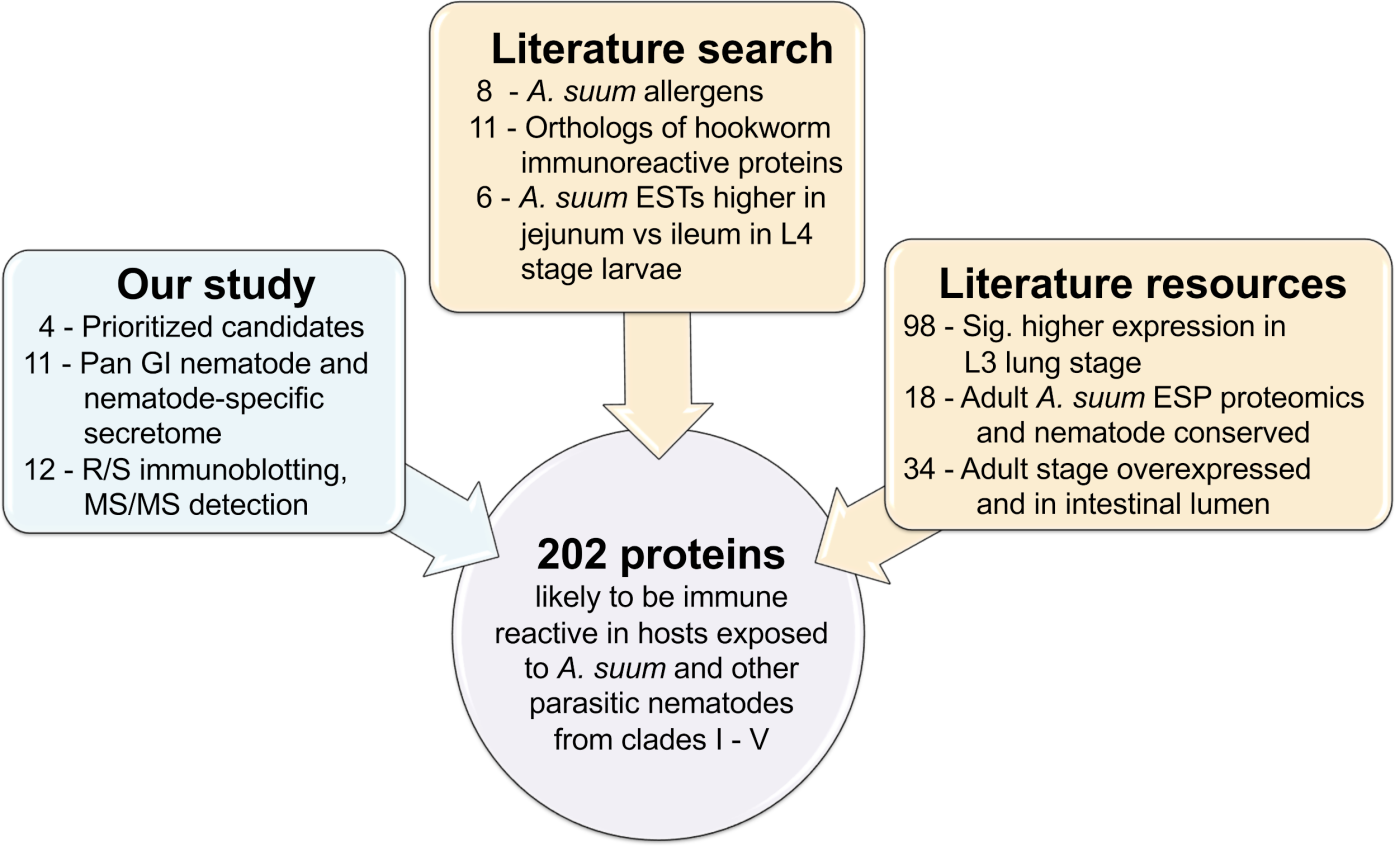
Overview of the *A. suum* protein prioritization process for selection on the protein array.

Overall, this prioritized database of antigenic OPFs (**Supplementary Table S2**) utilizing genomic, transcriptomic, and proteomic data spanning multiple nematode species, presents a valuable resource and approach to logically evaluate nematode-specific vaccine candidates with conservation across species. We also utilize this OPF database for the prioritization of additional candidates (**Figure 2B**, described below) and to select immunogenic candidates for the protein array (**Figures 1C**, **4**; described below). We have demonstrated that the three prioritized candidates do indeed exhibit cross-reactivity to other species even at 100-fold dilution of the antigen, and demonstrate the potential for using computational properties in generating targets for vaccination protocols. The approach and the database provided are novel resources for future research.

### 2.5 Additional protein prioritization based on immunogenic proteins in *T. suis*

Additional experimental evidence based on E/S products identified in *T. suis* was used to intersect candidate OPFs from the bioinformatic approach to identify and prioritize additional immunogenic proteins for downstream testing (**Figure 2B**; **Supplementary Figure S2**). A series of 2D gels were generated using culture derived fluorescent labelled parasite E/S antigens from either *T. suis* 28-day larvae or adults, then blotted and immune screened using sera from pigs infected with *T. suis* that were either resistant or susceptible to the development of adult worms (Applied Biomics, Hayward, CA; **Figure 2B**, **Supplementary Figure S1B**). Results identified 95 distinct spots, 51 of which reacted more strongly with 28-day larval E/S than adult E/S (**Supplementary Figure S2A**). The bias towards stronger reactivity to larval versus adult E/S proteins was preferred to select targets that may induce host protection to early developing parasitic stages of the infection rather than fecund adults that increase the potential for environmental contamination and additional infection of susceptible hosts. In a second phase of the study, IgG1 and IgG2 immunoblotting was performed using sera from pigs infected with *T. suis* and classified as resistant (cleared infection 52 days after experimental inoculation with infective eggs) or susceptible (>100 adult worms; **Supplementary Figure S2B**). Anti-*T. suis* and *A. suum* IgG1 antibody responses are induced more rapidly in infected pigs than IgG2 antibody response (Kringel et al., 2015). Comparing the two experiments, 30 spots comprising 34 *T. suis* proteins were selected for Mass Spectrometry (MS) proteomics testing. Of these, 19 reacted only with serum from resistant pigs and belong to 10 OPFs in the computational prioritization (**Figure 2**). These OPFs were prioritized according to the following criteria: (i) at least two of the GI nematode proteins contain putative secretory signals; (ii) the *T. suis* gene member is overexpressed in the 28-day larval stage; and (iii) the *T. suis* protein is detectable in 28-day larvae by MS proteomics. The proteins in these OPFs therefore represent 28-day larval proteins that are part of the E/S products, expressed at levels sufficient to be detected in the whole-worm proteomics dataset, and immunogenic in resistant hosts.

The three *A. suum* orthologs corresponding to these criteria are: (i) GS_08513, an actin binding protein conserved across nematodes (and no hosts), overexpressed in the *A. suum* intestine (Rosa et al., 2014) and detected by proteomics in the *A. suum* intestine (Rosa et al., 2015); (ii) GS_20415, a “Niemann-Pick C2” (cholesterol binding) protein (K13443) that was identified in the *A. suum* pseudocoelomic cavity (Rosa et al., 2015); and (iii) GS_04373, a “transthyretin-like” (TTL; IPR001534) protein, that possesses a nematode-specific function previously detected in the E/S products of many parasitic nematodes (Saverwyns et al., 2008), and found to be “by far” the most immunogenic protein among *H. contortus* E/S products (Yatsuda et al., 2003). The gene was also overexpressed in the *A. suum* head (Rosa et al., 2014) and detected by proteomics in the *A. suum* pseudocoelomic fluid (Rosa et al., 2015), but unfortunately this protein could not be cloned for further downstream testing. Based on this analysis, the *T. suis* proteins D918_06572 (GS_08513 ortholog) and D918_04417 (GS_20415 ortholog) were used in downstream immunization studies but are referred to by their *A. suum* ortholog IDs for consistency with array results and the other immunized proteins.

Overall, between the computational and experimental approaches (**Figures 1A**, **2**), five immunogenic protein candidates were identified based on a wealth of available “-omics” data and were expressed in bacteria for downstream experimental testing and validation. This combined forward and reverse genetics approach highlights the utility of the computational database for supplementing experimental research to prioritize targets and improve workflows.

### 2.6 The antibody-protein array revealed immune activation following immunization with the five-protein cocktail

**Figure 5:**
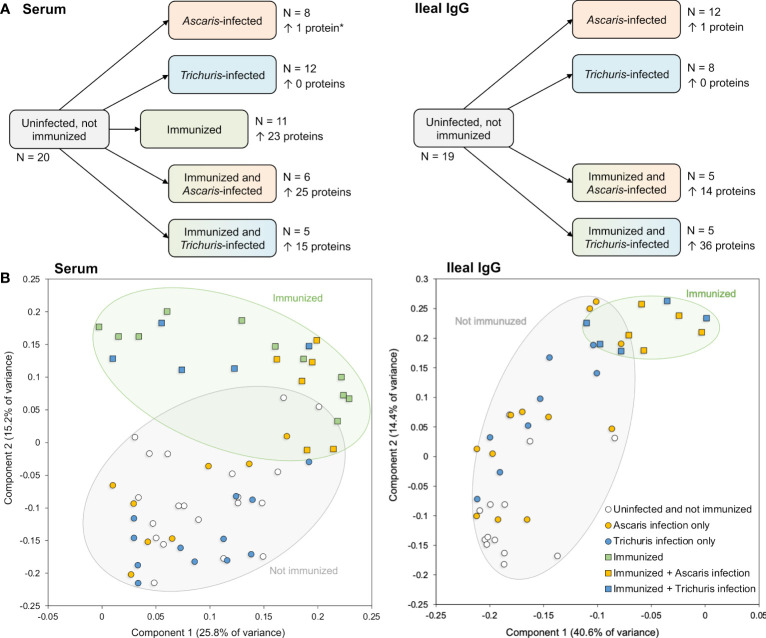
Overview of samples collected for analysis with the protein microarray to detect antibody production. **(A)** Sample sets including the number of samples, and the number of significantly differential detected proteins in each pairwise comparison (vs the uninfected and not immunized sample set). *The single protein in this comparison passed all filtering criteria except the local FDR threshold but was included for comparison to the Ileal IgG. **(B)** Principal components analysis (PCA) plots for each sample based on protein detection levels across all proteins with positive detection values.

A total of 202 recombinant *A. suum* proteins (including the five used for immunization) were used to generate a protein-antibody array and were prioritized according to our computational and experimental results and known proteins of interest and datamining datasets from the literature (including known *Ascaris* allergen proteins (Caraballo and Acevedo, 2011), orthologs of immunoreactive *N. americanus* proteins from a previous protein antibody microarray (Tang et al., 2014), adult *A. suum* E/S products detected by proteomics (Chehayeb et al., 2014) and adult *A. suum* intestinal lumen proteins, detected by proteomics (Rosa et al., 2015); **Figures 1C** and **4**; see methods for details, and **Supplementary Table S4** for accession information for all data collected from other studies. Sera samples were collected from pigs designated as a parasite infection cohort, consisting of pigs that were inoculated with a trickle infection of either *A. suum* or *T. suis* and another group given a mature primary infection with *T. suis* for 54 days, and a second immunization cohort consisting of pigs that were immunized with the five protein antigen cocktail three times over six weeks and bleed three weeks later followed by a challenge infection with either *A. suum* or *T. suis* and a subsequent bleed at either 27 (*Ascaris* challenge group) or 37 (*Trichuris* challenge group) days after the challenge infection. The timing of the last sera collection was at the time of necropsy and was designed to assess protective immunity from immunization that would provide detectable numbers of fourth stage *A. suum* and *T. suis* larvae prior to spontaneous host expulsion which would obscure a measure of acquired immunity from the immunization. Sera samples were also taken from pigs that were unimmunized and uninfected and served as a group for control comparisons (**Figure 1B**). Intestinal ileal fluid samples were also collected at necropsy from all cohorts except for the immunized only group. **Figure 5A** shows the sample groups and sample sizes used for statistical comparisons, after outliers were removed (see methods). The *A. suum* protein microarray (Antigen Discovery Inc, Irvine CA) was utilized to detect antibody responses in pigs immunized with the cocktail of cloned *A. suum* proteins, and to subsequent infections with *A. suum* or *T. suis* alone or after challenge infection of immunized pigs. All raw and processed array data have been deposited in NCBI’s Gene Expression Omnibus (GEO) (Edgar et al., 2002; Barrett et al., 2013) and are accessible through GEO Series accession number GSE234301. Antibody responses in each of the immunized/infected samples were compared to the uninfected sample cohort (**Figure 1D**). Several filters were applied in order to call a protein significantly and differentially detected: (i) based on output from Significance Analysis of Microarrays (SAM) (Tusher et al., 2001), both the Q value and local FDR values had to be ≤ 0.05 to ensure strong statistical support; (ii) the average detection level of the protein in the immunized/infected sample group had to be ≥ 10% above the median of the negative control probes on the array; and (iii) the fold change between the immunized/infected samples and the uninfected/unimmunized samples had to be ≥ 1.25 which is greater than the average fold change value plus the standard deviation of the fold change values across all proteins (i.e.,1.238) to ensure sufficient difference between the groups. Collectively, all three filters enabled confidence in the detection of positively reacting proteins on the array. All *A. suum* protein annotations, detection values and differential detection statistics for the array are provided in **Supplementary Table S5**.

Principal component analysis (PCA) clustering based on probes with an average value greater than the negative control probe showed that immunized groups clustered away from the unimmunized groups, regardless of *Ascaris* or *Trichuris* infection status (**Figure 5B**). This separation was supported by a statistical analysis of Pearson correlation values calculated between each sample pair based on the probe intensity data; In the serum, the average Pearson correlation between uninoculated and inoculated samples (0.0997) was significantly lower than the average correlation within inoculated and uninoculated samples (0.2811; *P* = 6.2x10^-63^, two-tailed T-test with unequal variance). The same was seen among the ileal IgG samples (average Pearson correlation = 0.267 and 0.478, respectively; *P* = 2.4x10^-19^). This indicates that the immunization with the five-protein cocktail stimulated immune responses in the pig host. Additionally, as seen on the PCA plot (**Figure 5B**), among the immunized serum samples, the *Ascaris-*infected samples clustered more closely with each other than they did with *Trichuris*-infected samples (average Pearson correlation = 0.628 and 0.341, respectively; *P* = 1.8x10^-6^), but the *Trichuris*-infected samples were as distant from each other as they were from the *Ascaris*-samples (*P* = 0.926). This indicates an overall more consistent serum immune response with *Ascaris* than with *Trichuris*, however these comparisons were not significant in the ileal IgG samples.

No substantial changes in antibody production were observed in the unimmunized pigs that were infected with worms. This finding is supported by the differential protein detection across groups in both serum and ileal fluid IgG (**Figure 5A**), with no proteins found to be significant in the *Trichuris*-only infection, and only one single protein (GS_03310) found to be differentially detected with *Ascaris* infection in the ileal IgG (although this difference was not significant when screened with serum). GS_03310 is an adult stage-overexpressed, *Ascaris*-specific secreted protein with no other functional annotation.

### 2.7 Immunogenic parasite proteins prioritized by the protein array analysis included known nematode allergens

**Figure 6:**
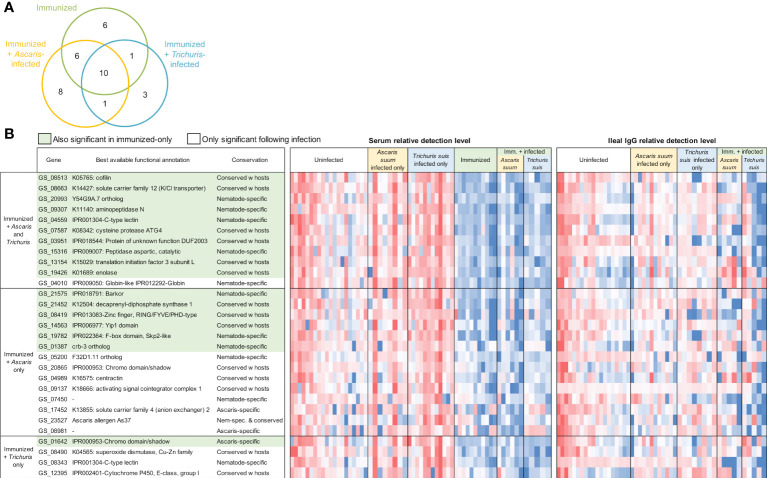
Differentially detected proteins among the immunized samples in the serum (vs uninfected + unimmunized samples). **(A)** Venn diagram showing the number of proteins significantly differentially detected between each of the three comparisons in the serum. **(B)** Proteins significantly differentially detected in the immunized and infected samples. Red shading indicates the proteins also significant in the immunized-only cohort. Relative detection level is calculated according to the Z score of the normalized protein detection levels for each protein. ‘Best available functional annotations included KEGG annotations, Interpro domains, and orthologs of *C. elegans* genes (in that order) and ‘conservation’ is based on the OPF analysis.

**Figure 7:**
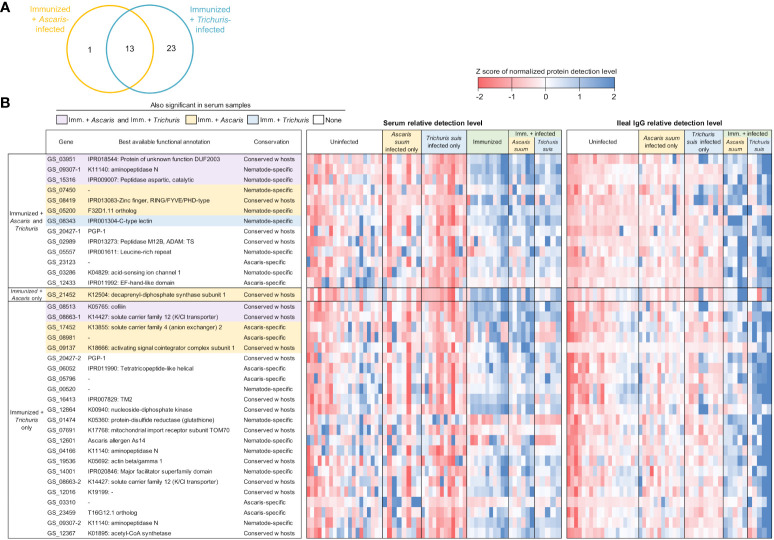
Differentially detected proteins among the immunized samples in the ileal IgG (vs uninfected + unimmunized samples). **(A)** Venn diagram showing the number of proteins significantly differentially detected between each of the three comparisons in the serum. **(B)** Proteins significantly differentially detected in the immunized and infected samples. Purple shading indicates proteins that are also significant in both the immunized + *Ascaris* and immunized + *Trichuris* serum comparisons, yellow shading indicates proteins that are also significant in the immunized + *Ascaris* serum comparison, and blue shading indicates proteins that are also significant immunized + *Trichuris* serum comparison. Relative detection level is calculated according to the Z score of the normalized protein detection levels for each protein. Best available functional annotations include KEGG annotations, Interpro domains, and orthologs of *C. elegans* genes (in that order) and conservation is based on the OPF analysis.

In the immunized and infected samples, between 14 and 36 A*. suum* proteins were found to be differentially detected (*P* ≤ 0.05) in each of the five comparisons (**Figure 5A**). In the serum, 23 proteins were detected significantly higher after immunization only, 25 were significantly higher with immunization followed by *Ascaris* infection and 15 were higher with immunization followed by *Trichuris* infection (**Figure 6A**); 10 proteins were significantly higher in all three comparisons (The top 10 proteins listed in **Figure 6B**). One of the eight elevated proteins found only in immunized and *Ascaris-*infected pigs was the allergen As37 (GS_23527), a highly immunoreactive L3 *A. suum* protein (Tsuji et al., 2002) that exhibits conserved intestinal expression across and specific to nematode species (Wang et al., 2015). In the ileal fluid IgG samples, 14 proteins were significantly higher in the immunized and *Ascaris* infected samples. Among these, seven overlap the proteins from the same comparison in the serum, and the probability is significantly low for this level of overlap to be due to chance (*P* = 8.2x10^-5^ binomial distribution test). Thirty-six proteins were higher in the immunized and *Trichuris*-infected samples (**Figure 7A**), six of which overlap the same comparison in the serum (*P* = 0.015 compared to random chance, binomial distribution test). Finally, 13 proteins were higher in both comparisons (listed first in **Figure 7B**). As14 (GS_12601), which when coupled with cholera toxin B induces protective immunity in mice against *A. suum* (64% reduction in larvae recovery) (Tsuji et al., 2001), was significantly higher in the immunized and/*Trichuris*-infected pigs. Across all comparisons, no proteins were found to be significantly lower following immunization and/or infection.

**Figure 8:**
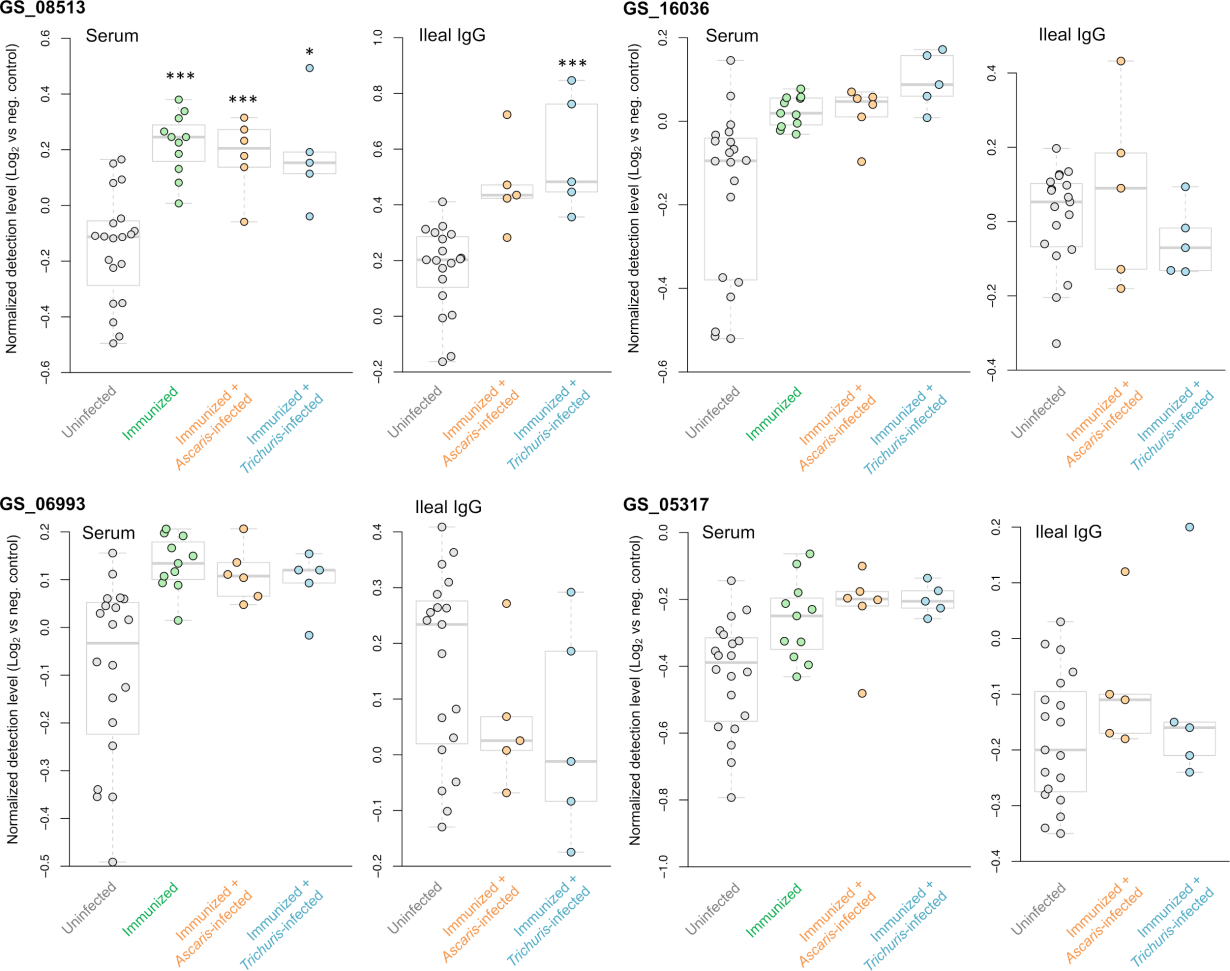
Differential detection of the four proteins used for the immunization that were tested on the protein array (out of five total immunized). Results are shown for the serum and ileal fluid (vs uninfected + unimmunized samples). Normalized detection levels represent the Log_2_ value of the fluorescence detection, relative to the median value of the negative control probes on the array. *** Q value ≤ 0.001, * Q value ≤ 0.05.

From the five *A. suum* proteins used in the immunization protocol, the relative detection of the four that were successfully added to the protein array are shown in **Figure 8**. The actin-binding cofilin protein GS_08513 prioritized in the second prioritization approach (**Figure 2B**) was detected significantly higher in four of the five immunized comparisons (all except the Ileal fluid IgG *Ascaris*-infected cohort). The other three proteins (the same from the ELISA experiments; **Figure 3**) showed higher detection with immunization in each of the three groups in the serum comparisons but did not meet the significance thresholds used for the analysis. This effect seems to be due primarily to high variability in the detection level among the uninfected serum samples.

**Figure 9:**
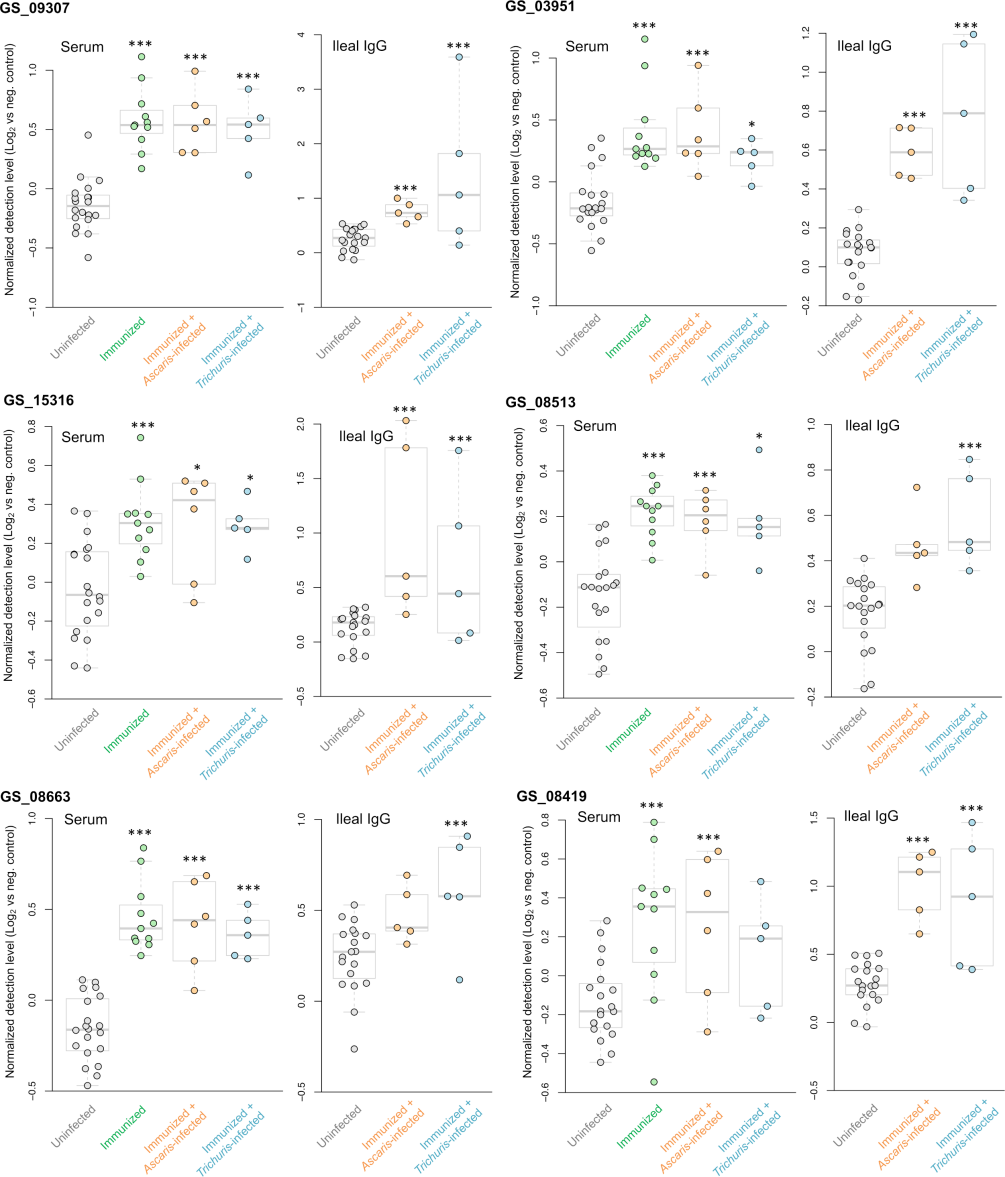
Differential detection of additional proteins of interest among the immunized samples in the serum and ileal fluid (vs uninfected + unimmunized samples). Normalized detection levels represent the Log_2_ value of the fluorescence detection, relative to the median value of the negative control probes on the array. Three proteins (GS_09307, GS_03951 and GS_15316) are significantly differentially detected in all five immunization experiments. Three proteins (GS_08513, GS_08663 and GS_08419) were significantly differentially detected in four out of the five of the immunization experiments. *** Q value ≤ 0.001, * Q value ≤ 0.05.

Three *A. suum* proteins were significantly higher in all five immunized comparisons (**Figure 9** and listed first in **Figure 7B**). The first, GS_09307, is a nematode-specific aminopeptidase N protein and an ortholog of an immunoreactive protein described in the previous *N. americanus* protein antibody microarray (Tang et al., 2014). There is precedence for aminopeptidases as vaccines because another aminopeptidase (H11) has been prioritized as a strong vaccine candidate in the parasitic nematode *H. contortus* (Roberts et al., 2013). Further, an aminopeptidase was identified as a human allergen in *Anisakis simplex* (a fish nematode parasite that causes allergic reactions when consumed) by proteomic analysis (Faeste et al., 2014). GS_09307 was also detected in the peripheral intestinal membrane (Rosa et al., 2014) and in the E/S products (Chehayeb et al., 2014) of *A. suum* by proteomics, The second protein, GS_03951, was overexpressed in lung-stage L3 of *A. suum* and is a nematode-conserved intestinal family protein (Wang et al., 2015); however to date, it lacks additional functional annotation. The third protein, GS_15316, has been detected in the intestinal lumen of *A. suum* by proteomics (Rosa et al., 2014) and was annotated as an aspartic peptidase and an ortholog of *asp-2* in *C. elegans.* The *N. americanus* ortholog of *asp-2* is a promising vaccine antigen for human hookworm infection, with effectiveness in reducing hookworm burden in vaccinated dogs (Bethony et al., 2005). These three proteins represent the best newly identified candidates for future immunization testing using *A. suum* proteins.

Finally, antibody binding to three additional *A. suum* proteins were significant in four of the five immunized comparisons (**Figure 9**): (i) GS_08513 (described above); (ii) GS_08663, a solute carrier family 12 (potassium/chloride transporter) member 4/5/6 protein that is a nematode-conserved intestinal family protein (Wang et al., 2015) and an ortholog of *C. elegans kcc-2*; and (iii) GS_08419, a WD40/YVTN repeat protein which was overexpressed in lung-stage L3 of *A. suum* and an ortholog of *C. elegans wdfy-2*.

Overall, the protein array data showed that immunization with the five-protein cocktail significantly induced antibody immune responses in pigs, both alone and when followed by nematode infection. These responses were more intense than nematode infection alone over the same time period. The dataset generated also identified specific nematode proteins which are strongly immunogenic across several immunization and infection cohorts (with *A. suum* and *T. suis*), including orthologs of known nematode allergens such as aminopeptidase and *asp-2.*

### 2.8 Conclusions

The benefits of including an effective vaccination component into an integrated control strategy to reduce parasitic nematode infection in livestock and humans is obvious, but the development of modern effective molecular vaccines in this area has been disappointing. This is undoubtedly due to the numerous parasite derived products produced by these complex metazoan organisms undergoing dramatic morphological changes and parasitic migratory behavior within the host that confounds a quest for a “silver bullet(s)” identification of meaningful vaccine targets. Novel approaches to both the selection of candidate proteins and models for immunization testing and evaluation of protective immunity that vary the dose, timing, adjuvant enhancement properties, routes of exposure, and other features are needed. The current study combined both a bioinformatics-based and experimental based approach to select five parasitic nematode-derived phylum-conserved immunogenic proteins with the highest potential for becoming GI nematode vaccine targets with efficacy across many species. The inclusion of *A. suum, T. suis* and *H. contortus* in the analysis provided a context for testing conserved nematode E/S antigens in mice that more conveniently address aspects of vaccine delivery and formulation than can be practically screened in large animal vaccination models. Testing of vaccine efficacy against the L3 lung stage of *A. suum* and *T. muris* in mice, as a surrogate for evaluating protective immunity against *Trichuris* species in other hosts including humans, is also well documented.

The five phylum-conserved target proteins selected and cloned for immunization testing in this study include three prioritized by a bioinformatics approach using available *A. suum* data, and two prioritized by the inclusion of experimental evidence in *T. suis* immunoblotting experiments, in addition to the bioinformatic prioritization (**Figure 1**; **Supplementary Figure S1**). Immunization with the five proteins induced antibody responses in pigs and activated immunity against several other parasitic nematode proteins including the *A. suum asp-2* ortholog, a protein which has been shown to be a strong vaccine target in *N. americanus*. PCA cluster analysis showed that the overall IgG antibody responses in both pig serum and intestinal ileal fluid were significantly altered by immunization but were unaccompanied by additional changes following a challenge infection with either *A. suum* or *T. suis*; no strong effects were observed in pigs that were infected but not immunized. This is somewhat perplexing given that the proteins selected for the immunization cocktail did show antibody binding with sera collected from *A. suum* and *T. suis* infected pigs (**Figure 3**). However, the sera used was from pigs that had been inoculated several times with infective eggs (trickle infection) or, in the case of *T. suis*, from pigs that had an adult worm infection of greater than 53 days post inoculation. The sera from pigs immunized with the five-antigen cocktail was collected at 27 days after a challenge infection with *A. suum* and 37 days after challenge with *T. suis*. These time periods were selected to recover fourth-stage larvae to assess protective immunity as a measure of vaccination efficacy and the limited worm development in the host after the challenge infection may not have expressed the antigens that were selected or expressed them in quantities insufficient to induce a memory response. In addition, this immunization protocol was also used to induce a protective response in C57Bl/6 mice that were subsequently challenged with *A. suum* and *T. muris* infective eggs and infective *H. polygyrus bakeri* third-stage larvae, but no significant protective immunity was induced (data not shown). Additional experiments with varying concentrations of immunization, worm burdens and timepoints should be considered in the future to fully evaluate protective responses with these antigens.

The 202 proteins that comprised the antibody-protein array, albeit small relative to the much greater possible number of secreted antigens from these parasites (277, 653 and 342 already identified for *A. suum*, *H. contortus* and *T. suis* respectively (Tritten et al., 2021)), provide a proof of principal that proteins selected based on bioinformatic, experimental, and literature-based criteria can be assembled and used for diagnostics and identification of further vaccine targets using sera and intestinal secretions from the large animal or human hosts of important parasitic nematode infections. Although the results provide identifications of immunogenic nematode parasite proteins, some false positives may be present due to non-specific binding and some false negatives or varying signal intensities may be present due to improper protein folding among the results or incorrect gene annotations on the draft nematode genomes used for recombinant protein production and for the bioinformatic prioritizations. In addition, it is difficult to correlate differences in antibody detection with outcomes in terms of infection clearance, since in some cases even with remarkably high antibody titers following vaccination, parasitic nematodes may persist and continue shedding eggs (as shown for both the Barbervax and Haemonchus-specific antibody vaccines in *Haemonchus contortus*-infected sheep (Kebeta et al., 2022)). However, Orthologous Protein Family clustering that includes comparisons with *H. polygyrus bakeri* predicted E/S proteins provides a context to evaluate the parasite protective capacity of identified proteins in laboratory mouse models of parasite infection.

The five target proteins used for immunization along with the additional downstream proteins that significantly activated immunity are targets for future study in host immunization testing. Further, the multi-omics database used to prioritize the targets (**Supplementary Table S2**) is a valuable resource for identifying additional proteins of interest. Overall, this research strongly supports future studies with the long-term goal of producing a successful effective vaccine to prevent parasitic nematode infection.

## 3 Methods

### 3.1 Orthologous protein family clustering

Orthologous Protein families (OPFs) across 15 deduced proteomes were defined using the Markov cluster algorithm available in the OrthoMCL package (Li et al., 2003; Fischer et al., 2011), with an inflation factor of 1.5. Species used in the OPF analysis included: *A. suum* (Jex et al., 2011) and *H. contortus* (Laing et al., 2013) genomes were obtained from their respective publications, *C. elegans* was obtained from WormBase (Harris et al., 2013), and *T. suis, H. polygyrus bakeri, D. viviparus, and O. ochengi* were based on genome versions used in the International Helminth Genomes Consortium (IHGC) publication (International Helminth Genomes Consortium, 2019). Genomes for relevant host species were obtained from ENSEMBL and GenBank (Harris et al., 2013; Sayers et al., 2023) and included *Homo sapiens*, *Canis familiaris*, and *Bos taurus, Canis familiaris, Mus musculus, Ovis aries*, and *Sus scrofa*. Genomes for two out-group species (*Drosophila melanogaster* and *Saccharomyces cerevisiae*) genomes were obtained from ENSEMBL (Cunningham et al., 2022). Versions and accessions for all genomes used are provided in **Supplementary Table S1**. The outgroup species were used to guide orthologous protein family clustering and more confidently identify proteins of interest shared among the four GI nematodes, while the host species allowed for the identification of nematode-specific proteins.

### 3.2 Computational secretome prediction

Nematode proteins across all species were categorized as putatively secreted if they contained no transmembrane domains (as predicted by Phobius (Kall et al., 2004)), and contained either a signal peptide for secretion (defined by Phobius) or a non-classical secretion peptide (defined by SecretomeP 1.0 (Bendtsen et al., 2004)), the same approach used for the International Helminth Genomes Consortium study (International Helminth Genomes Consortium, 2019). It is understood that secretion is a cellular event and that secretion from the cell may not translate to excretion/secretion from the parasite. Likewise, not all proteins that are secreted from the cell and/or parasite contain either a canonical signal peptide or a known non-classical secretion peptide.

### 3.3 Functional annotation and enrichment

Functional enrichment tests were performed for OPFs based on the gene members from the representative GI nematode *T. suis*. Interproscan (Jones et al., 2014) was used to identify Interpro domains in each gene in the *T. suis* genome (based on the predicted proteome). In addition, predicted proteins were searched against the KEGG database (Kanehisa et al., 2012) using KAAS (Moriya et al., 2007). GOSTATS v2.64.0 (Falcon and Gentleman, 2007) (which considers the hierarchical structure of GO) was used to determine significant functional enrichment among the proteins present in each set, with a *P* ≤ 0.01 significance threshold (after FDR population correction).

### 3.4 *Trichuris suis* gene expression quantification

RNA-seq datasets were retrieved from a previously published study (Leroux et al., 2018), including normalized relative gene expression levels (FPKM) from 10 (L2), 16 (L3), 17 (L3), 21 (L4), 28 (L4), 35 (Early L5), 42-day old (L5, adult) worms, as well as differential expression data for genes significantly overexpressed in early larvae, 28-day larvae and adult worms compared to other life cycle stages (Leroux et al., 2018). GenBank Sequence Read Archive (SRA) accession numbers for each sample are provided in **Supplementary Table S4**.

### 3.5 Cloning and protein expression

Cloning and expression was performed using standard techniques. The sequences GS_06993, GS_05317 and GS_16036 were PCR amplified from cDNA using sequence specific forward and reverse primers that encompassed the mature protein and contained Sac I (forward) and Xho I (reverse) restriction sites for downstream subcloning (all primer sequences are provided in **Supplementary Table S3**). Translation stop sites were incorporated into all reverse primers. Amplified sequences were first cloned into pCR2.1-TOPO by TA cloning then transformed into DH5α cells for sequence verification. Validated sequences were restriction enzyme digested and subcloned into the pSUMO bacterial expression vector (Life Sensors) containing a polyhistidine tag and transformed into BL21 cells for protein production. Expression was performed using overnight cultures of 500mL of LB medium containing ampicillin (100 µg/ml) which was induced at OD = 0.7 for 5 hrs at 37°C with isopropyl β-D-1-thiogalactopyranoside (0.3 mM final). Pelleted cells were lysed with 1 mg/ml lysozyme, frozen overnight then sonicated. Because all clones formed inclusion bodies during production, the sonicated pellets were first washed 3X with 2% Triton X-100, solubilized in 8M Urea (made fresh) and batch purified by affinity chromatography using 2 ml of Ni-NTA. All mixtures were added to columns, washed with 6M urea (3X), then wash buffer containing 50 mM sodium phosphate, pH 8.0, 300 mM sodium chloride and 20 mM imidazole. Recombinant proteins were eluted with 5 ml of wash buffer containing 500 mM imidazole and 12 mM sodium lauryl sarkosine.

### 3.6 Animal antigen immunization and parasite infection

Experimental pig barrows were obtained from a pig farrowing facility at the Beltsville Agricultural Research Center, Beltsville, MD. Pigs were derived from boars from a four-way crossbred composite BX line (Duroc X maternal Landrace X terminal Landrace X Yorkshire) designed by scientists at the USDA/ARS/US Meat Animal Research Center, Clay Center, NE to be genetically similar to genetics in the commercial swine industry at the time they were born; the genetics of the gilts are predominantly of the BX composite line. Pigs were from a herd screened yearly for porcine reproductive and respiratory syndrome virus (PRRSV), influenza (H1N1 and H3N2), pseudorabies, brucellosis and intestinal worm parasites by the Veterinary Services Group at the Beltsville Agricultural Research Center and have been negative for these infections. They were individually housed in stalls with a non-absorptive concrete floor surface covered with rubber mats with *ad libitum* access to water and a nutritionally adequate corn/soybean-based diet. All animal experiments and procedures were conducted in accordance with guidelines established and approved by the Beltsville Area Animal Care and Use Committee under protocol 17-019. Pigs in the infection only cohort included 1) three pigs inoculated five times every 10 days with 10,000 *T. suis* eggs and bled 10 days after the last inoculation, 2) six pigs inoculated eight times every other day with 10,000 *A. suum* eggs and bled 25 days after the last inoculation, and 3) three pigs inoculated with 10,000 *T. suis* eggs and bled 53 days later (these pigs were worm free and considered as a resistant phenotype). The second group of six pigs in the vaccination cohort were immunized with 400µg of recombinant protein (80µg each of the five-parasite antigen cocktail in Seppic Montanide ISA 61 VG [200µg, for a ratio of 1:2 for antigen:adjuvant] which was injected subcutaneously). The pigs were immunized a second time four weeks later and again after two weeks later and followed by a challenge infection with either 10,000 infective *A. suum* or *T. suis* eggs. The *A. suum*-infected pigs were bled and euthanized 27 days after inoculation and the *T. suis* infected pigs 37 after inoculation to collect blood and ileal wash fluid (contents from the ileum were removed and spun at 10,000 rpm and the supernatant fluid decanted and frozen at -80°C until used) from the small intestine (3 - immunized*/A. suum* infected).

### 3.7 ELISA experimentation

The top three prioritized OPFs from *A. suum* proteins were identified, respectively, as: 1) GS_16036 - NADH dehydrogenase, conserved across nematode outgroups, detected by proteomics in the *A. suum* intestine as a conserved 11 amino acid predicted antigenic region; 2) GS_06993 - Heme-binding/oxygen-transport protein, conserved across and only in parasitic nematode species, conserved in a seven amino acid predicted antigenic region*;* and 3) GS_05317 - No functional annotation, where the *T. suis* ortholog was defined as parasitic-stage specific and over-expressed; contained a conserved four amino acid predicted antigenic region that was absent in *T. suis.* Using these recombinant antigens in an ELISA with swine infection sera from pigs infected with *A. suum* (a pool of sera from three pigs given a primary and secondary infection with *A. suum*), *T. spiralis* (a pool of sera from three pigs at 60 days after a primary infection with *T. spiralis*) or *T. suis* (a pool of sera from three pigs that had cleared an adult *T. suis* worm infection at 53 days after inoculation with infective eggs). Antigens in 0.1M sodium bicarbonate buffer (pH 8.6) were plated overnight onto ELISA plates (Corning Costar, MO) by serial dilution ranging from 50 ng/well to 5 ng/well in a final volume of 100 μL. The next day, swine antisera diluted 1:250, were incubated for 2hrs with the plated antigens previously blocked with 5% dried milk in PBS-tween buffer. After washing, the secondary antibodies (goat anti-swine IgG peroxidase; Kirkegaard & Perry Lab Inc, MD) diluted 1:1000 were added and incubated for an additional hr. followed by washing and incubation in peroxidase substrate (Sigma-Aldrich, MO). Plates were read at 405 nm. The results indicated positive reactivity between all three antigens and *A. suum* and *T. spiralis* infection sera; however, reactivity between *T. suis* infection serum and the three antigens was limited.

### 3.8 Immunoblotting experiments

*Trichuris suis* adult E/S and day 28 larval E/S were prepared as previously described (Leroux et al., 2018) and separated in 2D-DIGE according to methods previously described for separation of *Toxoplasma gondii* tachyzoite and bradyzoite proteins (Hill et al., 2011). Basically, the adult E/S proteins were labeled with CyDye fluorescent signals Cy2 (green) and the day 28 larval E/S with Cy5 (red) and equal concentrations of proteins from each stage were mixed and loaded on a 2D-DIGE gel for analysis (shared proteins between the stages resulted in a yellow spot) and co-separated by isoelectric focusing in the first dimension (pH 4–9), and SDS-PAGE on a single multiplexed gel in the 2nd dimension (Applied Biomics, Hayward, CA). Two-dimensional gels resolving *Trichuris* proteins were subjected to Western blot and was secondarily screened with sera from pigs infected (1:200 dilution) with *T. suis* that had cleared the infection at 52 days after infection (resistant sera) (Applied Biomics, Hayward, CA). Replicate blots were then treated with Horseradish peroxidase conjugated-goat anti-pig IgG1 or IgG2 (Sigma Chemical. St. Louis, Missouri).

30 spots from the gels were selected for mass spectrometry proteomics identification. Protein identification was based on peptide fingerprint mass mapping (using MS data) and peptide fragmentation mapping (using MS/MS data).

### 3.9 Mass spectrometry and database searching

MALDI-TOF MS and TOF/TOF tandem MS/MS were performed on an AB SCIEX TOF/TOF™ 5800 System (AB SCIEX, Framingham, MA). MALDI-TOF mass spectra were acquired in reflectron positive ion mode, averaging 4000 laser shots per spectrum. TOF/TOF tandem MS fragmentation spectra were acquired for each sample, averaging 4000 laser shots per fragmentation spectrum on each of the 10 most abundant ions present in each sample (excluding trypsin autolytic peptides and other known background ions). Both the resulting peptide mass and the associated fragmentation spectra were submitted to GPS Explorer workstation equipped with MASCOT search engine (Matrix science) to search a database consisting of *T. suis, T. muris* and *T. trichiura* proteomes, to maximize the number of peptide detections (due to potential missed genes on the *T. suis* genome). Searches were performed without constraining protein molecular weight or isoelectric point, with variable carbamidomethylation of cysteine and oxidation of methionine residues, and with one missed cleavage also allowed in the search parameters. Candidates with either protein score C.I.% or Ion C.I.% greater than 95 were considered significant. *T. trichiura* and *T. muris* proteins were matched to *T. suis* genes according to a BLAST search (with confidence E value E-50), to finalize the list of identified proteins. 2-D DIGE and protein identifications were performed by Applied Biomics, Inc (Hayward, CA).

### 3.10 Protein selection for protein array

Several criteria were used to select proteins of interest to include on the protein array including existing genomic, transcriptomic, and proteomic datasets, as detailed below. To reduce complications with cloning, proteins corresponding to genes longer than 3000bp were not included for prioritization (except for some of the proteins identified by immunoblot, the *Ascaris* allergens, and the *Necator* orthologs). Not all proteins were able to be successfully cloned for the array (due to the availability of full-length cDNA), so the numbers presented here are the final counts of proteins on the array, and not necessarily the total number of proteins in the entire dataset meeting the criteria. Also note that some proteins may be prioritized based on several criteria, but the counts only include each unique protein that was not prioritized in previous steps.

The first group of proteins included those that were prioritized based on the current study’s computational and experimental prioritization schemes (**Figure 2**). These included (i) The cloned genes of interest used to immunize pigs (4; GS_20415 could not be cloned for the array but was used in the immunization); (ii) Pan GI-nematode nematode-specific secretome proteins (10); and (iii) Detected in resistant but not susceptible host immunoblots (13; **Supplementary Figure S2**).

The second group of proteins were mined directly from protein lists from the literature and included (i) Known *Ascaris* allergen proteins (Caraballo and Acevedo, 2011), identified with BLAST hits (8); (ii) Orthologs of immunoreactive *N. americanus* proteins from a previous protein antibody microarray (Tang et al., 2014) (11); (iii) Adult *A. suum* E/S products detected by proteomics (Chehayeb et al., 2014) and GI-nematode conserved in our study (18); and (iv) Adult *A. suum* intestinal lumen proteins, detected by proteomics (Rosa et al., 2015) (13). Although proteins excreted in the uterine fluid are available from a proteomics dataset, these were not considered in prioritization since they are hypothesized to be present for maintenance of egg and 1^st^ larval stage viability rather than for their effects on the host (Chehayeb et al., 2014), and as such may be helpful for sterilization rather than the prevention of new infections. Accession information for all data collected from other studies is provided in **Supplementary Table S4**.

The third group of proteins was mined from re-analyzing available literature datasets to identify protein candidates, and included: (i) *A. suum* Expressed Sequence Tags (ESTs) differentially expressed between L4 stage (21-day) larvae in the jejunum (where they usually survive and develop to adult) vs the ileum (where they are usually expelled following host self-cure) (Morimoto et al., 2003) (6); (ii) L3 Lung-stage overexpressed *A. suum* genes (relative to both L3 Liver and L4/Adult (Jex et al., 2011), *P* < 0.01 in each comparison using DESeq2 (Love et al., 2014)) (98); (iii) Adult-stage overexpressed genes (relative both the L3 Lung and L3 liver stages (Jex et al., 2011), *P* < 0.05 in each comparison using DESeq2 (Love et al., 2014)) and computationally predicted to be secreted (21).

### 3.11 Protein array preparation, array probing, raw data acquisition and normalization

Briefly, the clone library was created through an *in vivo* recombination cloning process with PCR-amplified coding sequences from cDNA, and a complementary linearized expressed vector transformed into chemically competent *E. coli* cells was amplified by PCR and cloned into the pXI vector using a high-throughput PCR recombination cloning method. The cloning methodology is described in detail elsewhere (Davies et al., 2005). All the clones were sequenced (Retrogen, Inc., San Diego, CA), and the results matched the correct target for the selected genes.

From each clone, the corresponding protein was expressed using an *in vitro* transcription and translation (IVTT) system, the *E. coli* cell-free rapid translation system (RTS) kit (Biotechrabbit, Berlin, Germany), as previously described (Davies et al., 2005). Each expressed protein includes a 5′ polyhistidine epitope tag and a 3′ hemagglutinin (HA) epitope tag. After expressing the proteins according to the manufacturer’s instructions, translated proteins were printed onto nitrocellulose-coated glass AVID slides (Grace Bio-Labs, Inc., Bend, OR) using an ArrayJet Marathon Argus robotic microarray non-contact printer (ArrayJet, Roslin, UK). Each slide contained 16 nitrocellulose pads on which the expressed proteins along with controls were printed (this allowed sixteen samples to be probed per slide using sealed chambers that isolate the arrays). Microarray chip printing and protein expression were quality checked by probing random slides with anti-His and anti-HA monoclonal antibodies with fluorescent labeling.

Serum samples were diluted 1:100 and ileal fluid samples were diluted 1:5 in a 3-mg/ml *E. coli* lysate solution (Antigen Discovery, Inc., Irvine, CA) in protein arraying buffer (Maine Manufacturing, Sanford, ME) and incubated at room temperature for 30 min. Arrays were rehydrated in blocking buffer for 30 min. The blocking buffer was removed, and arrays were probed with pretreated samples using sealed, fitted slide chambers to avoid cross-contamination between arrays.

For IgG Ileal Fluid assay, the arrays were incubated overnight at 4°C with agitation, washed three times with Tris-buffered saline (TBS)–0.05% Tween 20, and incubated with Goat anti-Swine IgG (Bethyl Laboratories, Montgomery, TX) diluted 1:500 in blocking buffer at room temperature. Arrays were washed three times with TBS–0.05% Tween 20 and incubated with Cy5 Donkey Anti-Goat IgG (Jackson ImmunoResearch, West Grove, PA) diluted 1:200 in blocking buffer at room temperature, protected from light. Arrays were washed three times with TBS–0.05% Tween 20, three times with TBS, and once with water and then air dried by being centrifuged at 1,000 × g for 4 min and left overnight in a desiccator before scanning.

For serum samples, the arrays were incubated overnight at 4°C with agitation, washed three times with Tris-buffered saline (TBS)–0.05% Tween 20, and incubated with Rabbit Anti-Pig IgG (MilliporeSigma, Burlington, MA) diluted 1:500 in blocking buffer at room temperature. Arrays were washed three times with TBS–0.05% Tween 20 and incubated with Cy3 Goat Anti-Rabbit IgG (Jackson ImmunoResearch, West Grove, PA) diluted 1:200 in blocking buffer at room temperature, protected from light. Arrays were washed three times with TBS–0.05% Tween 20, three times with TBS, and once with water and then air dried by being centrifuged at 1,000 × g for 4 min and left overnight in a desiccator before scanning.

Probed microarrays (slides) were scanned using a GenePix 4300A high-resolution microarray scanner (Molecular Devices, Sunnyvale, CA), and an image file (.tiff) was saved for each array using GenePix pro 7 software. The signals in the scanned images were quantified using the Mapix software (Innopsys) autogridding feature. For this process, two input files are required: (i) a.gal file that defines the array and subarray layout, and (ii) the.tiff image file for an array. Once the autogridding is complete, the overlays of the mapped array, subarray, and individual spot locations are shown in the graphical user interface (GUI). If the automatic gridding fails to map to the correct positions, the mapping can be manually adjusted using the GUI. Once the gridding is confirmed to be correct, the array spots are quantified and saved to an output.gpr file. For each spot on the slide, the.gpr file contains the foreground intensity (median of pixels inside the circle defining the spot) and local background intensity (median of pixels just outside the circle defining the spot). The final raw intensity is the foreground intensity minus the local background intensity. The raw signals were automatically extracted and saved as.csv files in data matrix format, with array spots as rows and samples as columns, using R (http://www.R-project.org).

Initial processing of the raw array data was performed as previously described (Li et al., 2017). First, raw values were transformed using the base 2 logarithm. Next, the data set was normalized to remove systematic effects by subtracting the median signal intensity of the IVTT control spots for each sample. Since the IVTT control spots carry not only the chip, sample, and batch-level systematic effects, but also antibody background reactivity to the IVTT system, this procedure normalizes the data and provides a relative measure of the specific antibody binding versus the nonspecific antibody binding to the IVTT controls. With the normalized data, a value of 0.0 means that the intensity is no different than that of the IVTT controls, and a value of 1.0 indicates a doubling with respect to IVTT control spots.

### 3.12 Bioinformatic analysis of protein array data

Log_2_ relative fluorescence values (relative to the median of the negative control probes) were calculated for each protein and each sample (one sample per array). Principal components analysis (PCA) was performed using the “prcomp” package in R, using only the proteins with relative Log_2_ fluorescence >0 relative to negative controls. Array data is available in **Supplementary Table S5**. Some samples were noted to have high or low values across all proteins on the array, so a systematic outlier removal approach was employed to ensure consistency among replicates that removes samples that appear to have this technical issue. For each sample cohort, a Manhattan distance matrix was calculated. The average and standard deviation of the average Manhattan distance between sample pairs was calculated within the cohort, and any individual samples with an average Manhattan distance more than (average + standard deviation) for the cohort were flagged as outliers and removed from the analysis. This removed approximately 8.5% of all samples, but never more than one sample per cohort, and was performed equally for both uninfected and infected cohorts.

Following outlier removal, Significance Analysis of Microarrays (SAM, implemented with SAMR version 3.0) (Tusher et al., 2001) two-class unpaired comparisons were performed using the Log_2_ fluorescence values relative to negative control probes. SAMR was configured with array settings, the T-statistic, with no median centering, output local FDR values, and Log_2_ input parameters. Filtering for “significant” proteins was performed independently for each comparison described in **Figure 5A**, and included: (i) Based on output from SAM, both the Q value and local FDR values were required to be ≤ 0.05, (ii) The average detection level of the protein in the immunized/infected sample group needs to be ≥ 10% above the median of the negative control probes on the array (iii) and the fold change between the immunized/infected samples and the uninfected + immunized samples needs to be ≥ 1.25. Across all comparisons tested, the average fold change value for each protein was 1.047, and the standard deviation was 0.191. So, the 1.25 threshold was selected to ensure that the difference between the immunized/infected samples was more than the average plus standard deviation (1.238) of the differences across all proteins. All three of these filters ensure confident significant detection of proteins on the array, and the values for each of these criteria for each gene and each comparison are provided in **Supplementary Table S5**.

## Data availability statement

The array data presented in the study are deposited in the Gene Expression Omnibus (GEO) repository, accession number GSE234301 (https://www.ncbi.nlm.nih.gov/geo/query/acc.cgi?acc=GSE234301). Versions, repositories, accesions and links to all existing parasite and host genomes used in the study are provided in **Supplementary Table S1**. Statistics and descriptions for all orthologous protein families across all data types are provided in **Supplementary Table S2**. Descriptions and links to previous publications for publicly available transcriptomic, proteomic and protein-antibody array sources are provided in **Supplementary Table S4**, with the published supplementary table or accession in NCBI's sequence read archive (SRA) indicated for each dataset. Protein-antibody array descriptions and statistical analyses are provided in **Supplementary Table S5**.

## Ethics statement

All animal experiments and procedures were conducted in accordance with guidelines established and approved by the Beltsville Area Animal Care and Use Committee under protocol 17-019.

## Author contributions

MM, JU and DZ conceived and designed the experiments. VF, EB, DH, AZ, DZ and JU performed the experiments. AY, XL, AS and AO generated the microarray array and performed the related analytical processing of the data. BR and MM analyzed the data. BR, MM, DZ, JU wrote the manuscript. All authors contributed to the article and approved the submitted version.
