# Supplementary material for: Identification of broadly-conserved parasitic nematode proteins that activate immunity
Source: Front Parasitol. 2023 Aug 8;2:1223942. doi: 10.3389/fpara.2023.1223942 (PMC11731683; doi:10.3389/fpara.2023.1223942)
Supplement: Supplementary file 1 [file DataSheet_1.zip › Supplementary Figure S1.docx]

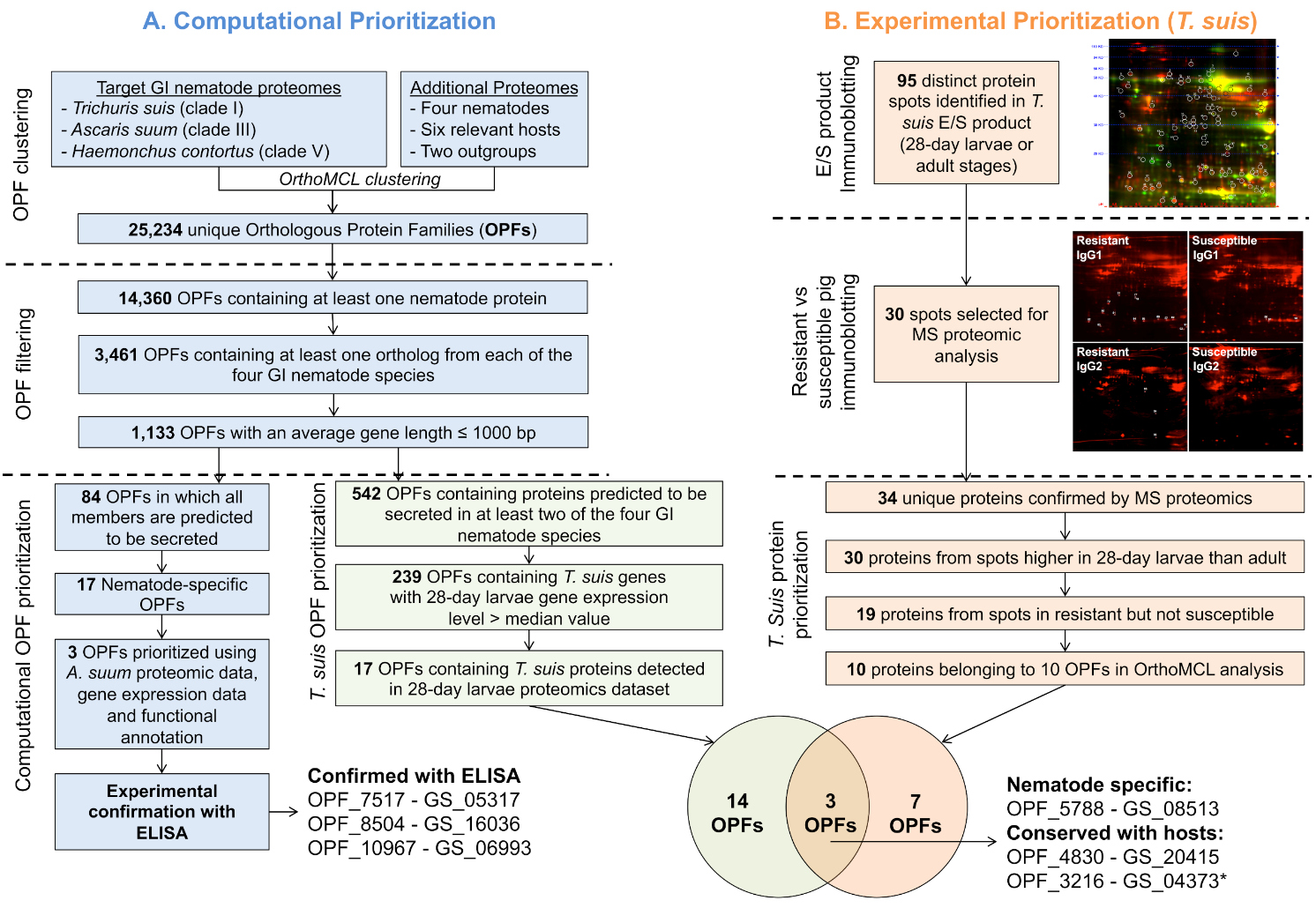


**Supplementary Figure S1:** A detailed overview of the orthologous protein family (OPF) prioritization process. *GS_04373 was not able to be successfully cloned for the downstream experimentation
