# Supplementary material for: Identification of broadly-conserved parasitic nematode proteins that activate immunity
Source: Front Parasitol. 2023 Aug 8;2:1223942. doi: 10.3389/fpara.2023.1223942 (PMC11731683; doi:10.3389/fpara.2023.1223942)
Supplement: Supplementary file 1 [file DataSheet_1.zip › Supplementary Figure S2.docx]

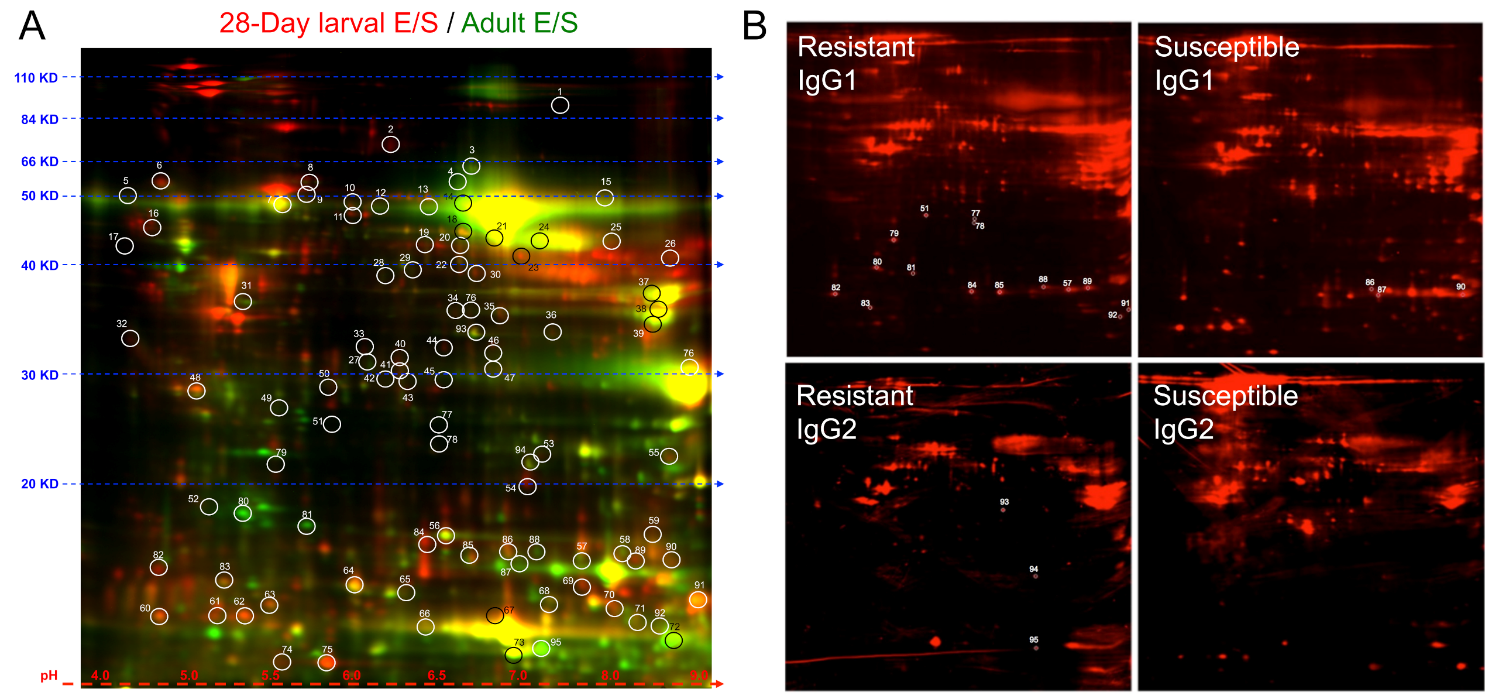


**Supplementary Figure S2:** Immunoblotting experiment results. (**A**) Immunoblotting performed on sera from pigs injected with 28-day larval and adult *T. suis* E/S products. 95 distinct spots are indicated. (**B**) IgG1 and IgG2 immunoblotting performed on sera from pigs infected with *T. suis* and classified as resistant (cleared infection) and susceptible (>100 adult worms).
