# Supplementary material for: Identification of broadly-conserved parasitic nematode proteins that activate immunity
Source: Front Parasitol. 2023 Aug 8;2:1223942. doi: 10.3389/fpara.2023.1223942 (PMC11731683; doi:10.3389/fpara.2023.1223942)
Supplement: Supplementary file 1 [file DataSheet_1.zip › Supplementary Table S3.docx]

## Supplementary Table S3: Forward (F) and reverse (R) PCR primers encompassing mature protein targets for ELISA experiments.

| Primer # | Primer name | Restriction Enzyme site  (underlined) | Primer |
| --- | --- | --- | --- |
| 1413 | GS_06993-F2 | Sst I | 5’ AAGAGCTCCATGGCGGCCTATTATGATGCGGAAG |
| 1414 | GS_06993-R2 | Xho I | 5’ AAACTCGAGTCAATACCATCCCCAAGTCTTCATG |
| 1417 | GS_05317-F1 | Sst I | 5’ AAGAGCTCCATGTCCGAATATTTGGGGACAA |
| 1418 | GS_05317-R1 | Xho I | 5’ AAACTCGAGTTAGCAATATCTTGGCATCGATCG |
| 1419 | GS_16036-F1 | Sst I | 5’ AAGAGCTCCATGGCCAGTGGGCAAAAGATCGCA |
| 1420 | GS_16036-R1 | Xho I | 5’ AAACTCGAGTCACTTGCTCGAACTATATCGATC |
